# Cancer‐associated thrombosis: Role of direct oral anticoagulants

**DOI:** 10.1002/ags3.12204

**Published:** 2018-09-05

**Authors:** Masataka Ikeda, Chu Matsuda

**Affiliations:** ^1^ Division Lower GI Department of Surgery Hyogo College of Medicine Nishinomiya Japan; ^2^ Department of Gastroenterological Surgery Graduate School of Medicine Osaka University Suita Japan

Because Trousseau first reported the relationship between cancer and thrombosis,^1^ it has been well known that thrombosis, especially venous thromboembolism (VTE), is frequently associated in patients with cancer. In Western countries, low‐molecular‐weight heparins (LMWHs) have been the standard of care for treatment of cancer‐associated thrombosis (CAT) after the CLOT trial.^2^ A meta‐analysis by Posch et al.^3^ demonstrated that LMWHs are superior to vitamin K antagonists (VKA) such as warfarin in reducing risk of recurrent VTE (RR = 0.60, 95%CI:0.45–0.79, *P* < 0.001) and have a comparable safety profile (RR = 1.08, 95%CI:0.70–1.66, *P* = 0.74). As VKA have drug interactions with anticancer agents such as 5‐fluorouracil (5‐FU), and their activity is highly dependent on food intake and stool conditions, which are very unstable during cancer treatment, precise control of VKA is difficult. In fact, in the Japanese population, VTE recurrence and associated major bleeding are highest in active cancer patients.^4^ Additionally, LMWHs cannot be used as a treatment for VTE in Japan.

Recently, it has been shown that direct oral anticoagulants (DOACs) have a noninferior efficacy on VTE recurrence and a superior safety profile compared to VKA treatment. Meta‐analysis of active cancer patients receiving either DOACs or VKA showed favorable trends for DOACs, but it was not significant (VTE recurrence: RR = 0.65, 95%CI:0.38–1.09, *P* < 0.10, major bleeding: RR = 0.72, 95%CI:0.39–1.35, *P* < 0.31).^3^ As these data originated from a subgroup analysis of large randomized control studies, we require a head to head comparison of DOACs to LMWHs as standard treatment for VTE in cancer patients.

Two randomized control studies comparing DOACs and LMWHs have recently been published: The Hokusai VTE Cancer Study^5^ comparing edoxaban and dalteparin, and the SELECT‐D Study^6^ comparing rivaroxaban and dalteparin. In both these studies, patients with active cancer with acute symptomatic or incidental VTE were randomly assigned to receive either DOACs or LMWHs. The primary outcomes were a composite of recurrent VTE or major bleeding during 12 months for the Hokusai VTE Cancer Study, and incidence of VTE recurrence over 6 months for the SELECT‐D Study. In the Hokusai VTE Cancer study, 522 patients received edoxaban and 524 patients received dalteparin. The primary‐outcome event occurred for 12.8% patients in the edoxaban group and for 13.5% patients in the dalteparin group (Hazard ratio, 0.97; 95%CI:0.70–1.36, *P* = 0.006 for noninferiority). Recurrent VTE occurred in 7.9% patients in the edoxaban group and 11.3% patients in the dalteparin group. Major bleeding occurred in 6.9% and 4.0% patients, respectively. In the SELECT‐D study, 203 patients were assigned to receive either rivaroxaban or dalteparin. The 6‐month cumulative VTE recurrence rate was 4% in the rivaroxaban group and 11% in the dalteparin group (Hazard ratio, 0.43; 95%CI:0.19–0.99). The 6 months of cumulative major bleeding rate was 6% and 4%, respectively. Based upon these two studies, the improved efficacy and safety of DOACs compared to LMWHs was proven and DOACs might be considered an alternative treatment to LMWH for VTE in patients with active cancer. Most frequently observed major bleedings were gastrointestinal (GI) bleeding in GI cancers in both studies. Bleeding may have occurred from the tumor itself or from the mucosa affected by antimetabolic agents such as 5‐FU. As the extent of mucositis caused by 5‐FU is different in Asian populations vs. the western populations, the bleeding events may differ in the Asian countries.

Direct oral anticoagulants are promising drugs for VTE treatment in patients with cancer; however, its safety in GI cancer patients remains unclear. Additional studies confirming its safety profiles in Asians are warranted.

## DISCLOSURE

Conflict of Interest: Author MI has Speakers' bureau and consulting or advisory role: Daiichi Sankyo and Bayer.
